# Role of connexins and pannexins during ontogeny, regeneration, and pathologies of bone

**DOI:** 10.1186/s12860-016-0088-6

**Published:** 2016-05-24

**Authors:** Lilian I. Plotkin, Dale W. Laird, Joelle Amedee

**Affiliations:** Department of Anatomy and Cell Biology, Indiana University School of Medicine, Indianapolis, IN 46202 USA; Roudebush Veterans Administration Medical Center Indiana, Indianapolis, IN 46202 USA; Department of Anatomy and Cell Biology, University of Western Ontario, London, Ontario N6A-5C1 Canada; INSERM U1026, Tissue Bioengineering, Université Bordeaux, Bordeaux, F-33076 France

## Abstract

Electron micrographs revealed the presence of gap junctions in osteoblastic cells over 40 years ago. These intercellular channels formed from connexins are present in bone forming osteoblasts, bone resorbing osteoclasts, and osteocytes (mature osteoblasts embedded in the mineralized bone matrix). More recently, genetic and pharmacologic studies revealed the role of connexins, and in particular Cx43, in the differentiation and function of all bone types. Furthermore, mutations in the gene encoding Cx43 were found to be causally linked to oculodentodigital dysplasia, a condition that results in an abnormal skeleton. Pannexins, molecules with similar structure and single-membrane channel forming potential as connexins when organized as hemichannels, are also expressed in osteoblastic cells. The function of pannexins in bone and cartilage is beginning to be uncovered, but more research is needed to determine the role of pannexins in bone development, adult bone mass and skeletal homeostasis. We describe here the current knowledge on the role of connexins and pannexins on skeletal health and disease.

## Backgound

Connexin (Cx) complexity: Cxs oligomerize to form hemichannels (connexons) that are transported to the cell surface where they dock with hemichannels from a contacting cell to form intercellular gap junction channels [1]. Channels typically cluster into crystalline structures known as gap junction plaques where they act to exchange numerous small molecules important in cell signalling [1]. To add to the complexity of connexin channels, undocked connexin hemichannels at the cell surface function to release small signaling molecules to the extracellular environment [2, 3]. Gap junction channels are even more complex as connexin subunits can form homomeric or heteromeric arrangements that dock across the extracellular space to form homo- or heterotypic channels. As an example, Cx43 has also been reported to form heterotypic channels with Cx40 [4, 5], Cx45 [6] and Cx46 [7]. Interestingly, these same connexins (Cx46, Cx45 and Cx43) are all found in the bone where they have the potential to create different types of channels with unique abilities to pass ions and small molecules as well as be regulated by pH, voltage, and posttranslational modifications [8].

In addition to their role as membrane channels, connexins have been shown to interact with intracellular structural and signalling molecules [9], adding yet another layer of complexity to their function. In particular for bone, it has been shown that Cx43 C-terminus domain interacts with β-arrestin [10], PKCδ [11], and α5β1 integrins [12, 13] in osteoblasts and osteocytes. Further, the Cx43 C-terminus domain is required for the survival effect of bisphosphonates and parathyroid hormone, and to enhance osteoblast signaling and gene expression following FGF2 administration [10, 14, 15].

## Role of connexins on skeletal ontogeny

Global deletion of Cx43 results in perinatal death due to impaired cardiac function [16], precluding the possibility to investigate the role of Cx43 in the mature skeleton. However, early studies performed in embryos showed delayed ossification both in intramembranous and endochondrial bone [17]. Similar results were observed in a later study [18]. This phenotype was observed in cranial bones, as well as clavicles, ribs, vertebrae and limbs. Interestingly, at the time of birth both axial and appendicular skeleton are normal, and only the cranial bones retain abnormal mineralization [17]. The expression of the osteoblastic gene osteocalcin is reduced during embryonic life (days 18.5 and 19.5 post-coitum), whereas alkaline phosphatase and osteopontin levels are reduced early on but normalized or even increased by day 19.5 [18]. In addition, osteoblasts lacking Cx43, or expressing only one copy of the gene (Cx43^+/-^) isolated from newborn mice exhibit reduced expression of bone matrix proteins and reduced mineralization potential *ex vivo* [17]. However, bone length during development [18] as well as size and morphology of the growth plate at birth are not affected by Cx43 deletion [17].

Less is known about the role of Cx43 in other cell types during bone development. A study showed delayed mineralization of cranial bones at birth in mice lacking Cx43 in osteochondro progenitors, as well as in mice expressing the oculodentodigital dysplasia (ODDD) mutant Cx43^G138R^ [19]. On the other hand, mice with the deletion of Cx43 in osteoblast precursors do not exhibit mineralization abnormalities, as evidence by whole mount alizarin red and alcian blue staining of newborn mice [20], suggesting that Cx43 expression in earlier precursors is needed for proper bone mineralization. In the case of other connexins that have been investigated, global deletion of Cx37 does not lead to changes in skeletal mineralization at birth [21]. Moreover, even though Cx45 and Cx46 expression has been demonstrated in bone cells, their role on skeletal development has not been studied.

## Mouse models of connexin deficiency and the skeleton

As indicated above, mice with global Cx43 deletion die soon after birth, precluding the study of the adult skeleton. Absence of one Cx43 allele in mice expressing a floxed Cx43 allele Cx43^fl/-^ mice does not alter bone mineral density accrual or bone mass in adult mice, compared to Cx43^fl/fl^ mice [20]. To overcome the lethality of the Cx43 full knockout, and to study the adult skeleton, several models of tissue specific deletion of Cx43 have been generated [22]. These mice lacking Cx43 in cells of the osteoblastic lineage have helped to understand the role of connexins in the skeleton. The bone phenotype of mice lacking Cx43 in osteoblastic cells is more striking when the gene is deleted in early progenitors, and becomes less profound when it is deleted in more mature cells. Mice lacking Cx43 in osteochondro progenitors (using Dermo1-Cre) exhibit decrease bone mass and reduced bone length [19]; whereas mice in which the gene is deleted in committed osteoblastic cells (Col2.3 kb-Cre) also exhibit low bone mass and decreased cancellous bone volume, but not changes in bone length [20, 23]. Mice lacking Cx43 in mature osteoblasts (OCN-Cre) do not exhibit low bone mineral density or cancellous bone volume [24, 25], neither do mice lacking Cx43 in osteocytes (DMP1-8 kb-Cre) [26]. In spite of the difference in bone mineral density and the cancellous bone phenotype of these mice, they all share a cortical bone phenotype, with increased periosteal bone apposition and bone perimeter, enlarged marrow cavity and accumulation of osteoclasts on the endocortical surface of the femoral mid-diaphysis [19, 25, 26]. Further, a recent study has proposed a new role for Cx43 in osteocytes [27] mediating intracortical bone remodeling and osteocytic osteolysis, a process by which osteocytes remove the surrounding bone matrix [28].

A recent report using genetically-modified mice revealed the role of Cx43 channel function in osteocytes [29]. In this study, 2 transgenic mice were generated, one expressing a mutated Cx43 with impaired channel permeability and the other expressing a Cx43 mutant able to form functional hemichannels but unable to form gap junction channels. Mice without functional channels or hemichannels exhibit increased bone mass, whereas mice expressing a Cx43 able to form hemichannels were not different from wild type littermate controls. Further differences were found between these 2 transgenic animal models, recently reviewed [22].

Unlike Cx43 full knockout mice, Cx37 deficient mice survive until adulthood and exhibit increased bone mass due to defective osteoclast function [21]. As for bone development, the role of Cx45 and Cx46 in the adult skeleton has not been explored.

## Connexins and bone regeneration

Only a few studies reported the role of Cx43 in bone repair and tissue regeneration in fracture healing models. Using a close femur fracture model, a recent study, revealed that Cx43 is widely expressed in the callus one month post-fracture [30]. Further, bone and total volume of the callus, as well as the number of TRAP+ osteoclasts are decreased in mice lacking Cx43 in osteoblasts and osteocytes (Cx43^fl/fl^;OCN-Cre mice) after fracture, compared to littermate controls. Cx43^fl/fl^;OCN-Cre mice also exhibit decreased mineralization during healing, compared to control mice expressing Cx43 in osteoblastic cells. In addition, the mechanical properties of the newly formed tissue are altered in Cx43^fl/fl^;OCN-Cre mice.

The effect of age on bone repair after damage and mechanical stimulation also involves gap junctional intercellular communication (GJIC) and Cx43 activity. For example, reduced osteocyte density and Cx43 levels were observed in regenerated bone in aged animals, limiting the establishment of GJIC, altering bone formation and bone resorption, as well NO and PGE_2_ secretion [31].

In contrast*, in vivo* transplantation of Cx43-transduced bone marrow stromal cells (BMSC) within gelatin scaffolds resulted in a larger quantity of bone relative to control cells. Bone regenerated from BMSC exhibiting enhanced GJIC also showed a thicker cortex and a large amount of trabecular-like bone [32]. These data suggest that Cx43 establishes a signalling platform to improve cell to cell communication in 3-dimensional (3D) structures and may have a major impact in the design of cell-based tissue engineering strategies for enhancing bone tissue regeneration [33, 34]. Further, modulation of connexin channels might also improve cellular interactions in cell-free scaffolds, by improving the communication among host cells recruited to the 3D structures.

These data also provide new insights into the 3D approach for the establishment stimulation of cell to cell communication. Mesenchymal stem cells cultured in 3D matrices for bone tissue engineering express higher levels of Cx43 compared to 2D cultures in plastic culture dishes [35]. The 3D microenvironment modifies the distribution of cells cultured within the matrices and enhances the cellular contacts. Further, the spheroid organization of cells within scaffolds contributes to the increase in Cx43 expression and new bone formation in experimental models [35].

Osteoinductivity of calcium phosphate-based scaffolds is also likely mediated by Cx43 expressed by dental pulp cells [36]. These findings are expected to advance the design of future tissue engineering materials in which Cx43 could be used to activate bone cell differentiation and bone formation. In this context, a cell-permeant mimetic peptide, alpha connexin carboxyl-terminal peptide (αCT1), based on the carboxyl-terminus of Cx43, has been shown to elicit changes in gap junction organization and GJIC associated with upregulation of protein kinase C-mediated phosphorylation of Cx43 in cell systems other than bone. It has been demonstrated that this mimetic peptide reduces scar progenitor and promotes regenerative healing following skin wounding [37] and also augments corneal wound healing [38], suggesting that it could be used to enhance bone ormation in bone scaffolds.

## Connexin gene mutations and human disease

Early in the new millennium, germline mutations in the *GJA1* gene encoding Cx43 were found to be causal of oculodentodigital dysplasia (ODDD) [39]. Nearly all ODDD mutations are inherited in an autosomal dominant manner and cause syndactyly, camptodactyly, craniofacial abnormalities, enamel hypoplasia, cartilage anomalies that result in a thin nose and ophthalmic defects [39–45]. There are now at least 76 Cx43 (*GJA1*) mutations linked to ODDD [39–42, 45–67]. So far, 100 % of the ODDD patients harbor mutations in one of the *GJA1* gene alleles that encode Cx43 [61] but there are now autosomal mutations linked to Cx43 that do not cause ODDD but rather cranio-metaphyseal dysplasia (R239Q) [68] and sudden infant death (SID) (E42K, S272P) [69]. In addition, two recessive *GJA1* mutations (encoding R33X and R76H) have been reported [41, 42, 70]. Patients homozygous for the R76H mutant not only exhibit symptoms of ODDD but also Hallermann-Streiff syndrome denoted by a small stature, congenital cataracts, hypotrichosis, beaked nose, skeletal anomalies and teeth defects [42]. It is intriguing that specific Cx43 mutants cause different disease symptoms with variations in autosomal dominant or recessive inheritance and it is intriguing that nearly all mutants cause bone abnormalities.

## Cx43 gene mutations cause disease by different mechanisms

Many categories of disease-linked Cx43 mutants have been identified *w*hich include changes in connexin half-life, dysregulated pH and/or voltage gating and assembly defects that lead to loss- or gain- of channel or hemichannel function [60]. Of the documented mechanisms, one group includes mutants that assemble into gap junction channels (I130T [71–74]) but have known reductions in channel function. Another class of mutants includes those with altered intracellular trafficking, typically resulting in mutants being retained in the endoplasmic reticulum and/or Golgi apparatus (fs230, fs260 [75, 76]). Still other mutants fall into a class that have a gain-of-function where hemichannel (G138R [77]), or channel function is enhanced beyond what is observed for wild-type Cx43 (G143S). Finally, Cx43 mutants may be efficiently transported to the cell surface and assemble into gap junction plaques, but remain functionally dead (G21R [78]). Collectively, these findings suggest that specific mutations may exhibit distinct mechanisms of action that have direct bearing on the clinical presentation of ODDD.

Disease-linked mutants are co-expressed with wild-type Cx43 and may contribute to the overall level of Cx43-based GJIC if functionally active, or alternatively, inhibited wild type Cx43 function if functionally dead. For example, the I130T mutant exhibits ~20 % normal channel function when expressed alone [73] and, together with co-expressed wild-type Cx43, maintains Cx43-based GJIC at >50 % [72, 73, 79]. In other cases, the mutant may be dominant-negative to co-expressed wild-type Cx43 (G21R, fs260 [76, 78, 80, 81]) resulting in total Cx43-based GJIC to be <50 %. Since nearly all human cells co-express Cx43 along with other connexin family members, the mutants have the theoretical potential to exhibit transdominant negative properties on other connexins. This condition seems to be rare as the hearts of ODDD patients, for example, are rarely diseased. However, bone anomalies are common in ODDD patients where osteoblasts and osteocytes express Cx37, Cx45 and Cx46 [82–88] all of which can potentially interact with co-expressed Cx43.

## Cx43 in bone and cartilage

Bone development, remodeling and repair require the exquisite and coordinated activity of osteoprogenitor cells, osteoblasts, osteocytes and osteoclasts all of which express Cx43 that mediates both hemichannel function and GJIC [86, 87, 89, 90]. While Cx43 is by far the predominant connexin in cells of osteogenic lineage, Cx37, Cx45 and Cx46 have also been found [89]. Several reports using Cx43 knockout mice and conditional ablation of Cx43 from osteoblasts and osteocytes during early development have demonstrated excessive endocortical bone resorption together with periosteal enlarging resulting in reduced whole body bone mass together with cortical widening and thinning [20, 26, 91–93]. Connexins in cartilage are less well understood but Cx43 is again the predominant connexins in mesenchymal cells and chondrocytes [94–98] while Cx45, Cx32, and Cx46 expression have also been reported [99]. Collectively, these studies suggest that Cx43 plays an essential role in skeletal development. In addition, increasing evidence supports the notion that Cx43 also plays a key role during bone remodeling in aging, as its ablation has been reported to desensitize osteoclasts that typically become activated after the removal of mechanical load [100]. Further, the increase in GJIC in response to PTH and cholera toxin is diminished in cells from old (12-month-old) compared to young (4-month-old) rats [101]. We know that ODDD patients consistently exhibit craniofacial anomalies yet little information exists as to whether there are changes in long bones. Phenotypic evaluation of Cx43^G60S/+^ mice revealed thinner cortical bones, enlarged marrow cavity, decreased mineral density, a decline in trabecular bone volume and reduced overall mechanical strength [102]. Most recently, these mice were found to have higher levels of osteoprogenitor cells and greater osteoblast function leading to the up-regulation of bone sialoprotein and the receptor activator of NFκB ligand [103]. While young mutant mice had greater osteoclast number leading to osteopenia, this condition was self-corrected during aging [103]. In a second conditional mouse model of ODDD where the G138R mutant was introduced into cells of osteochondro lineage, skulls were found to be smaller and whole body bone mineral density was less as the mice suffered from cortical thinning [19]. Thus, while it is clear that Cx43 plays a key role in bone development and remodeling the mechanisms involved remain largely unknown and it has yet to be determined how these skeletal changes manifest during bone fracture and healing. In addition, we have little knowledge of how ODDD mutants affect hemichannel and gap junction channel status in cells of osteogenic lineage. Hemichannel function, in particular, is of considerable interest as several studies have shown that shear stress-induced opening of hemichannels allows the release of prostaglandins and ATP resulting in the activation of paracrine signaling pathways [89, 104]. The importance of these findings is enhanced by the fact that at least a few ODDD mutations (e.g., G138R) result in gain-of-hemichannel function [77].

Cx43 has been shown to be involved in the response of the skeleton to different insults. For example, mice lacking Cx43 in osteoblastic cells and subjected to ovariectomy, a well-known maneuver to mimic post-menopausal bone loss, does not lose bone mass 3 weeks after surgery, unlike littermate wild type controls [105]. However, bone mineral density in Cx43-deficient mice reaches similar values to those of wild type mice 4 weeks post-ovariectomy, suggesting that bone loss induced by lack of sex steroids is delayed, but not abolished, in the absence of osteoblastic Cx43.

In addition to its role in skeletal development and bone cell function, *in vitro* and *in vivo* evidence supports a role of Cx43 on bone acting stimuli. In particular, the survival effect of bisphosphonates and parathyroid hormone (agents used to treat osteoporosis and other bone diseases) on osteoblastic cells required Cx43 expression *in vitro* [10, 14]. The requirement of Cx43 for bisphosphonate survival effect has been confirmed *in vivo*, in mice lacking Cx43 in osteoblasts and osteocytes [24]. Further, the bone anabolic effect of intermittent parathyroid hormone administration is diminished in mice lacking Cx43 in osteoblastic cells [20].

Cx43 has also been involved in the effect of mechanical stimulation on the skeleton. In particular, mice lacking Cx43 in osteochondro progenitors, in osteoblastic cells, or in osteocytes exhibit an increased response to mechanical stimulation in bone [25, 26, 106]. Further, Cx43 deletion from osteoblastic cells attenuates bone loss induced by reduced mechanical forces [93, 107].

## Pannexins

Upon their discovery in the new millennium, pannexins (Panxs) gained instant attention as a possible new family of gap junction proteins due to their limited homology to invertebrate gap junction proteins [108]. The pannexin family of channel proteins consists of Panx1, Panx2 and Panx3. Through the use of a molecular toolkit it was discovered that members of the pannexin family are long-lived, channel-forming glycoproteins that function in ATP release [109–111]. While the proposed role of pannexins as molecular constituents of intercellular channels remains unlikely, there is general agreement that Panx1 forms large single membrane channels at the cell surface that serve a role in paracrine signaling [112]. For example, Panx1- [113] and Panx3-mediated [114] ATP release plays a role in calcium wave propagation which may involve their interplay with purinergic receptors [115–118]. ATP and UTP released via Panx1 channels also serves as “find-me” signals for clear apoptotic cells [110]. Signaling through Panx1 channels may also contribute to cell death and seizures under ischemic or epileptic conditions [119–121], lead to inflammatory bowel disease [116], promote melanoma disease progression [122] and even facilitate HIV-1 viral infection [123]. Panx3 has been linked to the proliferation/differentiation of keratinocytes [124], chondrocytes [125, 126] and osteoblasts [114, 125, 127]. Other studies highlighted the regulatory role of Panx1 and Panx3 in keratinocyte differentiation while Panx3 was found to be important in osteoprogenitor cells, chondrocytes and osteoblasts [124, 125, 127]. Amongst a variety of tissues, Panx1 mRNA and protein has been detected in the developing and mature cartilage and bone [109, 112, 128]. Panx3 has a more restricted distribution pattern in the body but widely found in skeletal tissue, including pre-hypertrophic chondrocytes and perichondrium osteoblasts [112]. In addition, a recent study showed that Panx2 is present in extracellular matrix vesicles obtained from mineralizing osteoblastic cells [129].

## Role of pannexins in skeletal tissues

It is now well established that Panx3 is expressed in cartilage where it may regulate chondrocyte proliferation and differentiation [125, 126]. This notion is supported by the up-regulation of Panx3 during terminal differentiation of chondrocytes [130, 131]. Panx3 was also found to be of critical importance in the maturation of growth plates in the chicken embryo. Interestingly the expression of Panx3 in N1511 and ATDC5 cells promoted differentiation of chondrocytes which was inhibited in Panx3 knockdown studies [126]. Chondrocyte differentiation was further linked to the reduction and in cAMP and ATP release [126]. Through what might be attributed to calcium waves and ATP release, Panx3 appears to govern osteoblast differentiation [114]. Consistent with our studies showing Panx3 expression in skeletal tissues and its regulation by the skeletal master transcription factor Runx2 [125], it is highly likely that Panx3 plays a key role in cartilage and bone development. The involvement of pannexin channels in acquired pathological conditions has only been reported in skeletal muscle atrophy [132].

Mice with global deletion of Panx1, 2 and 3 have been generated, as well as double Panx1/2 knock outs [133, 134]. All these mice are viable; however, the consequences of pannexin deletion on bone/cartilage phenotypes (or lack thereof) have not been reported, except in the case of Panx3 where it appears to play a role in osteoarthritis (see below).

## Connexins and pannexins in osteoarthritis

Osteoarthritis is a progressive disease of the joint affecting over 15 % of the world population [135]. This untreatable disease tends to affect the aging population as multiple joints experience articular cartilage degeneration which includes deterioration of the synovium, bone and ligaments localized to joints [136, 137]. Molecular mechanisms that govern this process are ill-defined but appear to include changes that result in aberrant hypertrophic differentiation of articular chondrocytes [138, 139].

### Role of connexins

Several studies over the last few years show a correlation between aberrant Cx43 expression and OA. In particular, Cx43 expression and the presence of gap junction plaques is increased in synovial lining cells isolated from the knees of patients with OA [140]; and Cx43 levels are elevated in the cartilage of the knees and femoral heads [99] and in the shoulders [141] of patients with osteoarthritis. In addition, Cx43 levels in osteoarthritic cartilage correlate with the expression of several pro-inflammatory and catabolic factors [141]. Further support for a role of Cx43 in cartilage is provided by the protection of inflammation and joint destruction by silencing Cx43 in a model of rheumatoid arthritis in rats [142]. In addition, a recent proteomic study showed that the profile of Cx43-interacting proteins changes in primary chondrocytes isolated from patients with OA, compared to normal donors [143].

### Role of pannexins

Panx3 has been shown to have a potentially important role in OA as it was found to be upregulated in the reticular cartilage of rats surgically treated to accelerate the onset of OA [144]. A few years later this notion was further supported by Iwamoto and colleagues [126] as they found that knockdown of Panx3 blocked hypertrophic chondrocyte differentiation. As an extension to these studies the first global and cartilage-specific Panx3 null mice were generated and subjected to OA onset by destabilization of the medial meniscus surgery [134]. In both cases, mice lacking Panx3 developed less severe OA. Not surprisingly when human biopsies from OA patients were assessed and compared to OA in mice, both exhibited high levels of Panx3 suggesting that Panx3 was instrumental in the development of OA [134]. Current studies are underway by this same team to determine if Panx1 serves any role in OA onset and progression. Nevertheless, these studies suggest that Panx3 may be a potential target in the treatment of OA.

## Conclusion

**Fig. 1:**
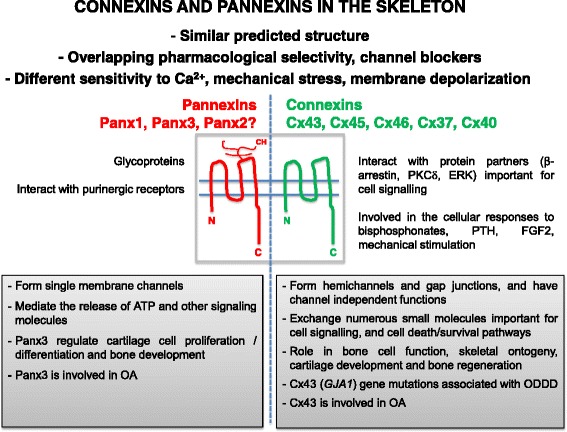
Figure summarizes the current understanding on the role of connexins and pannexins in the skeleton. OA, osteoarthritis; ODDD, oculodentodigito dysplasia; CH, carbohydrate chain

Mounting evidence supports a central role of Cx43 for skeletal development, maintenance, and response to bone acting stimuli (Fig. 1). Further, mutations of the Cx43 gene in humans are linked to ODDD, a disease with skeletal manifestations. On the other hand, the role of pannexins in the skeleton is beginning to be uncovered, and pannexins seem to have a more relevant function in cartilage than in bone.

Genetically-modified animal models have provided fundamental information on the role of connexins and pannexins in skeletal tissue. However, similarities and differences between connexins and pannexins still remained enigmatic. Basically, ascribing a particular function to connexins *vs.* pannexins on the effects of bone acting stimuli and for skeletal disease remains a difficult problem plagued by the overlapping pharmacological selectivity between channels, compensation by the others isoforms, methodological differences in assessing channel function, and genetic alterations associated with transgenic mouse models [145]. Therefore, better tools are needed to understand the role of these channels in bone and cartilage. Furthermore, a fundamental task for future research is to find compounds that specifically modulate the actions of connexins or pannexins, allowing their use as pharmacological agents to treat diseases of the skeleton.
